# Born Too Soon: Care for small and sick newborns, evidence for investment and implementation

**DOI:** 10.1186/s12978-025-02032-y

**Published:** 2025-06-23

**Authors:** Sarah Murless-Collins, Veronica Chinyere Ezeaka, Nahya Salim Masoud, Karen Walker, Natasha R Rhoda, William Keenan, Steve Wall, Zulfiqar A Bhutta, Pablo Duran, Olufunke Bolaji, Karen Edmond, Gagan Gupta, Joy E Lawn

**Affiliations:** 1 London School of Hygiene & Tropical Medicine, London, United Kingdom; 2 College of Medicine, University of Lagos, Lagos, Nigeria; 3 Department of Paediatrics & Child Health, Muhimbili University of Health and Allied Sciences, Dar Es Salam, Tanzania; 4 Council of International Neonatal Nurses, Sydney, Australia; 5 Department of Paediatrics, School of Child and Adolescent Health, Division of Neonatal Medicine, University of Cape Town, Cape Town, South Africa; 6 International Paediatric Association, Chicago, United States of America; 7 Department of Global Health, Save the Children, Washington DC, United States of America; 8 SickKids, Toronto, Canada; 9 Pan American Health Organization, Buenos Aires, Argentina; 10 Department of Paediatrics Federal Teaching Hospital, Nigeria & African Neonatal Association, Ido-Ekiti, Ekiti, Nigeria; 11 Department of Maternal, Newborn, Child and Adolescent Health and Ageing, World Health Organization, Geneva, Switzerland; 12 United Nations International Children’s Emergency Fund (UNICEF) Headquarters, New York, United States of America

**Keywords:** Small and sick newborn care, Neonatal, Premature, Family-centred care, Systems change

## Abstract

**Progress:**

Over the past decade, the world has made policy progress for newborns including the first global Sustainable Development Goal (SDG) target 3.2 (< 12 neonatal deaths per 1000 live births) and the Every Newborn Action Plan (ENAP). However, gaps remain for investment and action, especially for babies born too soon, too small, or who become sick. An estimated 20–30 million newborns have life-threatening conditions requiring hospital care each year. Annually, approximately 2.3 million newborns die during the neonatal period, the majority being preterm. A further 1 million newborn survivors are estimated to have long-term disabilities.

**Programmatic priorities:**

To achieve SDG 3.2 by 2030, we need to accelerate four-fold. The shift to 80% of births in health facilities creates opportunities for impact, for both maternal and newborn care. Increased coverage and quality of high-impact newborn interventions is urgently needed to reach SDG targets. Most neonatal deaths and disabilities are preventable through an evidence-based package for small and sick newborn care (SSNC), with greatest impact seen in preterm babies—particularly through respiratory support and kangaroo mother care—while placing families at the centre of care. SSNC scale-up requires addressing ten core components, defined by WHO/UNICEF, based on a health systems approach: political commitment and leadership; financing; human resources; appropriate infrastructure; equipment and commodities; robust data systems and use of data for action; referral systems; linkage with high-quality maternal care; family and community involvement; and post-discharge follow-up. Specific focus is required for fragile conflict settings, accounting for 25% global births but 39% global newborn deaths.

**Pivots:**

More ambitious investment in high-quality, family-centred care for vulnerable newborns can give a high return of between US$ 9−12 for every US$ 1 invested. Accelerating implementation requires diverse stakeholders, including political leaders, bureaucratic and technical leadership in country, professional societies, civil society, the private sector and importantly from families and communities. Cross-country collaboration and strengthening capacities of low- and middle-income countries to address gaps in newborn care are essential for innovations to reach high-burden, conflict-affected, and marginalised populations. Integrating newborn care follow-up into wider child and family care systems is crucial to ensure newborns not only survive but also thrive.

## Plain Language Summary

**Progress:**

In the last decade, policies for newborns have moved forward, but there are major gaps in action, especially for babies born too soon and others with complications. Every year, tens of millions of newborns face life-threatening conditions, especially for those who are preterm, or born too soon. Around 2.3 million newborns die in the first 28 days, with prematurity as the main cause. Another million survive with long-term disabilities.

**Programmatic priorities:**

To meet targets, notably the Sustainable Development Goal 3.2 (under 12 neonatal deaths per 1,000 live births) and the Every Newborn Action Plan, there’s an urgent need to improve newborn care. The shift of births to health facilities (now 80%) provides a new opportunity for saving more lives. Most neonatal deaths can be avoided through small and sick newborn care which requires ten core health system components: political leadership, funding, trained staff, infrastructure, medical supplies, reliable data systems, referral networks, maternal care integration, family involvement, and follow-up after discharge.

**Pivots:**

More investment is needed to ensure all newborns have access to high-quality care, including those in conflict zones or marginalised groups. Over the next decade, countries can go faster and learn together to innovate and improve for newborn care. Ensuring follow-up care for developmental outcomes is foundational for newborns to reach their full potential.

## Key Findings

**Progress:**

• Over the past decade, the world has set new policy targets for newborn survival, including the first ever globally agreed neonatal survival target but gaps remain for investment and programmatic action, especially for babies born too soon, too small, or who become sick.

• Globally, the place where babies receive care has shifted, with >80% of births now occurring in health facilities.

• An estimated 20-30 million small and sick newborns have life-threatening conditions that require inpatient care in hospitals each year, half of whom are preterm.

**Programmatic priorities for inpatient newborn care:**

• Quality improvement and availability of inpatient newborn care is urgently needed now to reach SDG target 3.2 (<12 deaths per 1000 live births) and Every Newborn Action Plan targets.

• Most major causes of neonatal death can be prevented by adopting a health systems approach to the scaling up of small and sick newborn care (SSNC) in countries. This requires ten core components: political commitment and leadership; financing; human resources; appropriate infrastructure; equipment and commodities; robust data systems and use of data for action; referral systems; linkage with high-quality maternal care; family and community involvement; and post-discharge follow-up systems.

**Pivots:**

• Invest more ambitiously in SSNC to ensure equitable universal access to high-quality, family-centred services, including in areas of conflict and for marginalised populations.

• Implement high-quality, family-centred care with widespread involvement and support from families, communities, professional societies, politicians and business communities.

• Innovate through multi-country learning, including sharing of existing tools and development/improvement of technologies and systems for SSNC in all contexts, especially high-burden settings.

• Integrate family-centred care and follow-up for developmental outcomes into existent systems newborn, linking with maternal care, to ensure that the newborns who survive also thrive.

## Purpose

This paper focuses on 20−30 million babies born each year estimated to require hospital care to treat life- threatening conditions, most of which currently do not receive care or receive it too late [1]. Every minute counts for these vulnerable newborns, and the quality of care can determine their survival, influence their brain development, and affect their entire life-course and contribution to society.

Collectively this series of seven papers was developed from the report “*Born Too Soon: A decade of action on preterm birth”* [2]. The report aligned to a campaign for preterm birth, linked to slowing progress for maternal and newborn health and stillbirths, noting the lack of progress for preterm birth as a key factor. Content included collation of new data, literature reviews and case studies, organised into three themes: (1) ***progress*** notably in the last decade; (2) programmatic ***priorities*** from the evidence; and (3) ***pivots*** to accelerate change in the decade ahead. The first paper summarises the definitions and terminology.

## Progress

### The past decade: what has moved for newborn care?

**Fig. 1:**
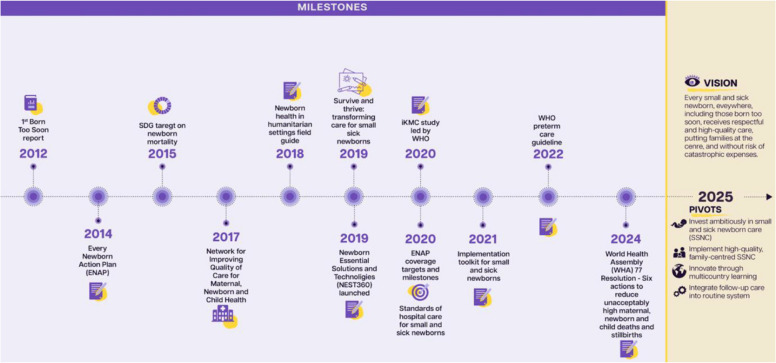
Newborn health: timeline of progress over the past decade and vision for the next decade

The world has made progress over the last decade, with new urgency to prevent newborn deaths, particularly among babies born preterm [3]. Some milestones are depicted in Fig. 1, chief among them the Every Newborn Action Plan (ENAP), adopted in 2014 by 194 countries [4]. Subsequently, the Sustainable Development Goals (SDGs) included the first ever target for reducing newborn mortality to < 12 deaths per 1000 live births. The Global Strategy for Women, Children’s and Adolescents’ Health also endorsed these targets and included one for stillbirth prevention (i.e., < 12 stillbirths per 1000 live births by 2020)[5]. Most importantly, over 100 countries have now committed to newborn survival targets and action plans, with leadership from national governments, the United Nations (UN) and many partners. A specific coverage target for small and sick newborn care (SSNC) was included as part of the joint Every Woman Every Newborn Everywhere (EWENE) targets for 2020–2025 to give impetus and monitor district level operationalisation [6]. A detailed section has been included in the ENAP-EPMM progress tracking report to track progress on different components needed for subnational scale-up [6].

Building on this policy momentum, the “Survive and Thrive: transforming care for every small and sick newborn” report presented a call to action [1]. Importantly, it included the term “newborns born too small who become sick”, together with preterm newborns, as the population most vulnerable to death and long-term disability. It also a underlined that major impact is possible with over 740,000 deaths averted per year before full neonatal intensive care. In 2020, ENAP coverage targets were updated to include specific targets for every district (or equivalent unit) to have at least one functional small and sick newborn unit [7]. UN standards and technical guidelines have been published, bringing together the evidence for this package [8, 9].

Despite policy progress, an estimated 2.3 million newborns died within their first 28 days of life in 2022 [10], translating to 6400 newborn deaths each day. Prematurity remains the leading cause of under-5 mortality [10]. Almost all newborn deaths (98%) occur in low- and middle-income countries (LMICs), and 78% occur in sub-Saharan Africa and Asia. The “four Cs” (i.e., conflict, climate change, COVID-19, and the cost-of-living crisis) further threaten hard-won gains and heighten risks for already vulnerable newborns [11]. The “fifth C” of complacency also needs to be acknowledged, as this is a bigger threat than the other “four Cs” combined, and perhaps the greatest barrier to progress for SSNC scale-up. Further challenges to progress over the last decade have been discussed in detail in paper 1 of this supplement [11].

### The next decade: what are the gaps for newborn care?

There is still a dearth of investment in newborn health. Overall, US$ 15.9 billion of donor funding is spent annually on reproductive, maternal, and child health. Newborn health is relatively new and was barely mentioned before 2005 [12]. In 2019, just 10% of these donor disbursements mentioned the world “newborn”, mostly in the phrase Maternal, Newborn and Child Health (MNCH), despite around half of under-5 child deaths occurring during the neonatal period [13]. Most importantly < 1% of these donor disbursements included a high-impact neonatal intervention such as breastfeeding, kangaroo mother care, etc. Domestic government spending is the majority in most countries but is harder to track consistently across countries. Generally, countries that have made more rapid progress in neonatal mortality rate (NMR) reduction, report to have invested more [10, 12].

Although multiple evidence-based interventions are available to improve survival, geographical disparities in implementation coverage and quality persist. Innovations exist, but gaps in stable distribution and training, unreliable supply chains, lack of skilled workers, and out-of-pocket payments at point of care present continuing barriers to roll-out. Despite pregnant women accessing health facilities, mortality rates for both mothers and newborns remain high due to inadequate quality of care, as health systems struggle to deliver consistent, high-standard services. Care for newborns and their mothers is often siloed, and there is an urgent need to better integrate newborn care into maternal, referral, and follow-up services to operationalise a systems approach across the continuum of care.

### Poised for programmatic acceleration

The world is at a tipping point for newborns and our next generation. To achieve the vision and goals set out in the ENAP and national SDG targets, more action is urgently needed. Despite policy progress for newborns, investment and implementation of evidenced-based care has been slower. However, many countries are now poised for programmatic action due to two substantial shifts in newborn care over the past decade.

#### Shift 1—Mortality transition requiring changing interventions

**Fig. 2:**
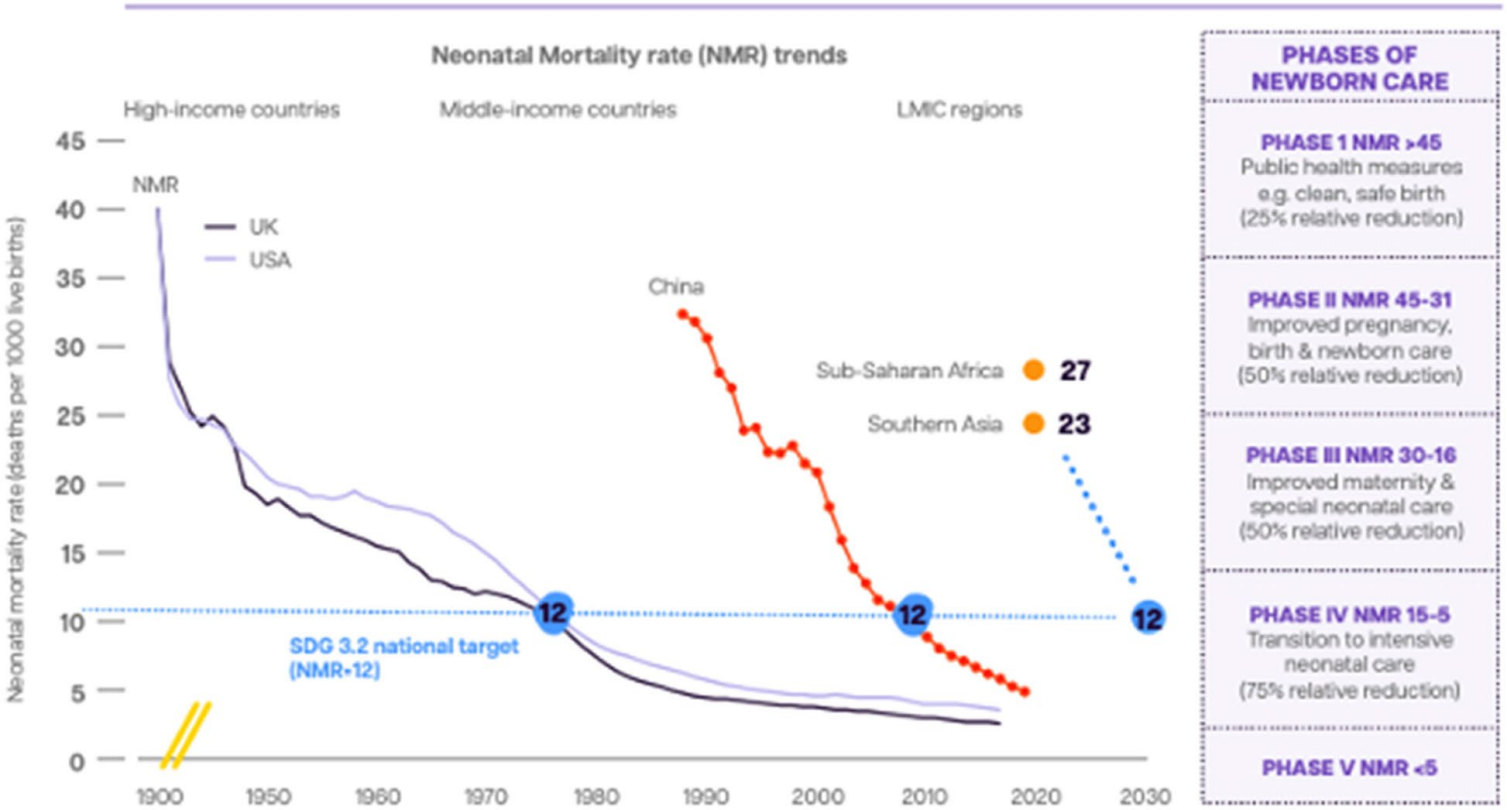
Historically informed neonatal mortality rate (NMR) reductions by phases of care *Adapted from* [15]

Countries have reduced their NMR over five phases, and this applies both to historical changes in the UK and USA and more recent rapid changes in, for example, China (which has the fastest progressing NMR reduction in the world) (Fig. 2). Given progress, there are now few countries that are in the highest mortality band (phase I), and these are mostly affected by conflict. In these highest mortality settings, many births are at home and there are more deaths due to infection. Different solutions are needed especially through primary care (e.g., family planning, maternal immunisations, water, sanitation and hygiene, infection prevention and control, etc.). Most of the world’s births and neonatal deaths are in the 48 countries with an NMR of 16–30 deaths per 1000 live births (phase III). These countries have already established most of the simpler programmes available. To accelerate mortality change and meet the SDG 3.2 target of 12 neonatal deaths per 1000 live births, the highest impact will be from small and sick newborn care (SSNC). Maternal health care (especially obstetric care) is also crucial to prevent hypoxic birth injuries, but few countries have successfully reduced preterm birth, so care of these babies will be needed everywhere. Countries that invested in systems change, notably their nursing and midwifery workforces, have achieved sustained reductions in maternal and newborn mortality [14].

#### Shift 2—Place of birth and of care

The second notable shift is the place of care. Facility-based births have increased over the last decade: more than 80% of the 134 million annual births worldwide now occur in a health facility [16]. A woman and a baby’s chances of surviving and thriving are largely determined by the services available and delivered at the place of birth. The increased coverage of facility births therefore presents an important opportunity to improve quality of care with families at the centre [17].

##### Caring for vulnerable newborns in highest mortality, conflict-affected settings

Many of the highest mortality countries are in conflict zones[18] and one in every ten preterm babies is born in a fragile context [19]. A recent Lancet Series highlighted the lack of high-quality data from conflict-affected areas –particularly on newborn care compromising delivery of essential interventions [20]. Evidence-based guidance to improve newborn care in humanitarian settings does exist [21, 22], but weakened health systems and limited skilled health workers makes caring for small and sick babies in such settings a major challenge. Humanitarian systems are developing innovative solutions, including more flexible ways to bring care closer, such as mobile clinics and community-based care. Task sharing can be helpful, for example with community health workers and outreach workers. Engaging communities remains vital [23].

## Programmatic priorities

In many countries with high birth rates and NMR (Fig. 2), there has been a longer focus on maternity care and SSNC is newer. Investing in SSNC requires important health systems transformation. These changes need to also be accompanied by wider transformation of social, economic and legal institutions as discussed in paper 3, including important wider intersectoral change with novel approaches [24].

### What inpatient care is needed for small and sick newborns?

**Fig. 3:**
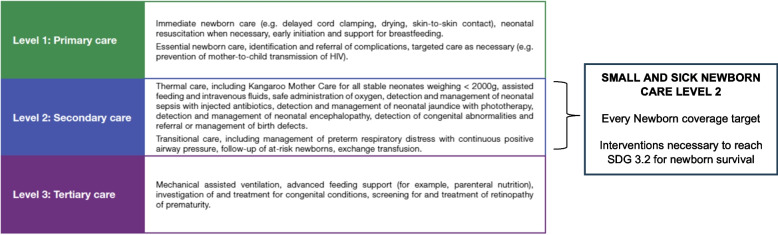
Standards of care and evidence-based interventions *Sources: WHO/UNICEF Survive and Thrive, 2018 *[1]* WHO, 2020 *[8]

For faster change in the majority of countries, the top priority is increased coverage of high-quality care for all admitted newborns –including preterm babies– with a package for SSNC made available at district level. The most common organisation of maternal and newborn care is a tiered system with three interconnected levels, mirroring primary, secondary and tertiary care at the population level and bringing care closer to home (Fig. 3). Every district, or equivalent subnational planning unit, needs a facility providing SSNC level-2, with families at the centre, follow-up care, and functioning referral services from and to level-1 and level-3 facilities [7]. This requires beyond a “one at a time” project approach, shifting to government-led systems for implementation.

Preterm babies are the most frequent admissions in most neonatal units in most countries, and the highest impact interventions for them that are part of level-2 care are respiratory support, notably Continuous Positive Airways pressure (CPAP) and Kangaroo Mother Care (KMC). Immediate initiation of KMC, even prior to stabilisation, has been found to reduce mortality in premature and small newborns if higher duration is reached [25]. Apnoea of prematurity is a common complication among preterm infants (< 37 weeks gestation). Access to caffeine citrate, started promptly especially is newborns < 34 gestation, is a proven safe and effective treatment to prevent apnoea of prematurity [9]. Caffeine availability is a challenge in the highest burden settings but can be overcome. Other evidence-based interventions such as surfactant become cost-effective at lower levels of NMR – for example surfactant was introduced in the USA and UK once the national NMR was under 5 (Fig. 2).

SSNC units must also be able to provide care for babies with other common causes of neonatal death such as birth complications, infections, congenital conditions and jaundice. The priority interventions are summarised by level in (Fig. 3). More specialised services available in tertiary centres are required to address the needs of extremely premature newborns (i.e., < 28 weeks), including advanced feeding support (e.g., parenteral nutrition), assisted/mechanical ventilation, and surgical services.

**Fig. 4:**
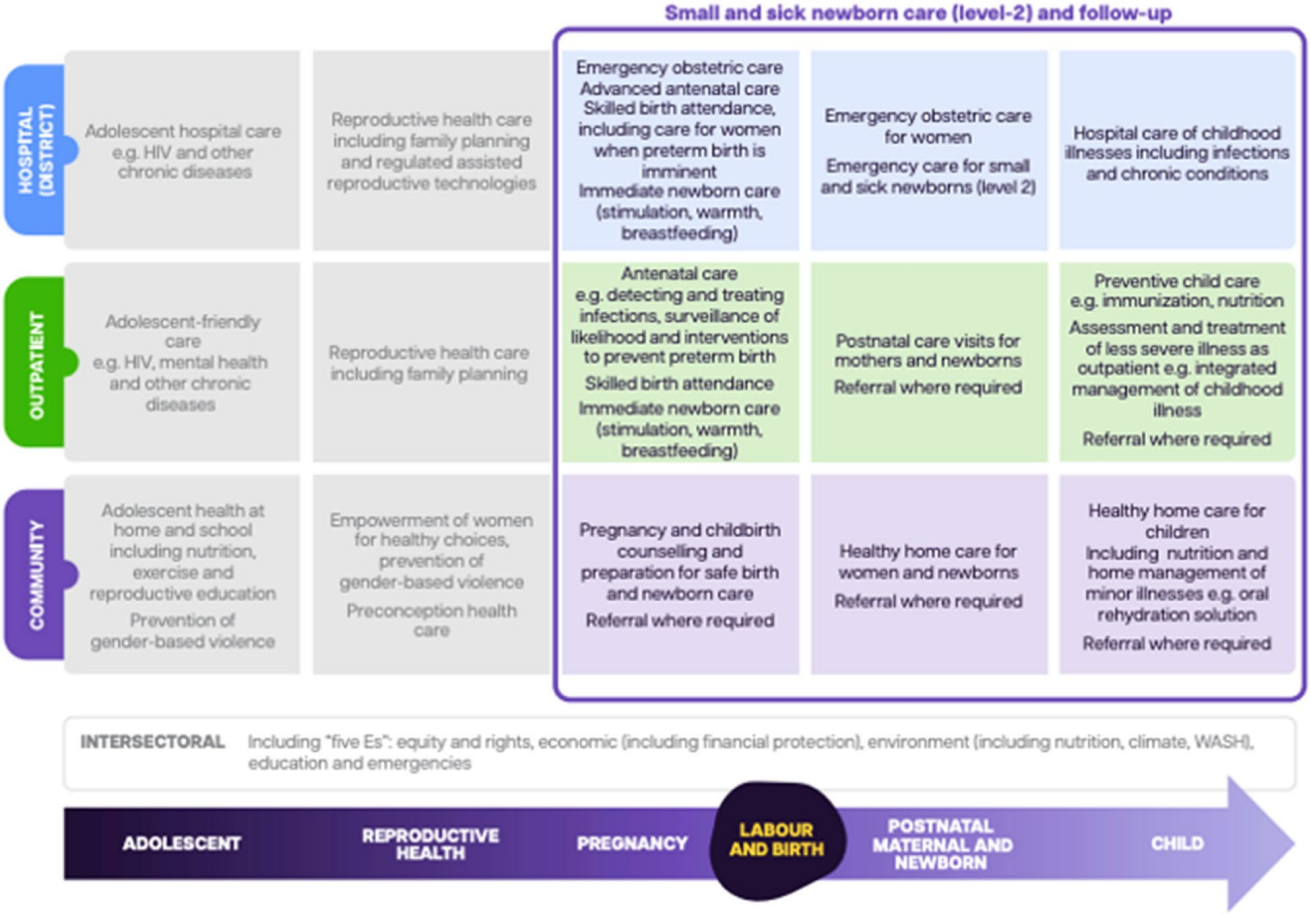
Continuum of care, with packages focused on small and sick newborn care (level-2) and follow-up care

For small and sick newborns to survive, minutes count, and their families must be able to quickly access the appropriate level of care within the health system without major distance or cost barriers. These levels need to be interconnected by communication and referral systems, and to function within the continuum of care for maternal, newborn and child health (Fig. 4).

It is important that the SSNC package is centred on families and has developmentally supportive care at the core from the start [1]. Globally, 13 million preterm newborns survive each year, 2.7% of them with moderate to severe impairments and at least 4.4% having mild neurodevelopmental impairments [1]. To optimise brain development and human capital, all newborns require family-centred, developmentally supportive care, minimising separation from parents and including the following components:

- Optimise nutrition: provide human milk; use cue-based, infant-guided feeding; involve parents in feeding to improve breastfeeding initiation and duration [26].
- Safeguard sleep: cluster care; provide care to coincide with sleep and wake cycles; minimise noise and light [27].
- Manage pain and stress: recognise signs of stress and pause intervention when possible; use positioning and boundaries to provide containment [28]. Maintain head in midline with limbs and trunk flexed and tucked; handle with slow, gentle movements; talk gently; provide support during transfers [29].
- Protect skin: maintain humidity during skin maturation; monitor susceptible skin/mucosal areas for breakdown; target wireless monitoring systems; promote skin-to-skin contact and massage; initiate breastfeeding early; position correctly [30].

Family engagement in inpatient care of newborns results in better health and development outcomes, improved patient and family experience, higher health worker satisfaction and better resource allocation. Maximising contact with parents, especially mothers, promotes bonding, breastfeeding, cognitive development and shorter hospital stays. Without support for parental participation, unintentional harm can occur to both newborns and families. Active family involvement requires context-specific adaptation and flexibility, and parental support groups/networks play a crucial role in driving change [17]. Empowerment of mothers/caregivers with knowledge and health education is vital. Data from ENAP-EPMM tracking in 2023 from 106 countries shows only 51% countries have a provision in policy/guidelines for engagement of families in the care of babies and in only 42% countries planning of level-2 SSNC units include provisions for the mother to stay with her baby in the newborn care unit.

Mothers, fathers and families need information and support when a baby is stillborn or when a newborn dies [31]. As with end-of-life care for all patients, newborns with untreatable conditions are entitled to a dignified and pain-free death. A newborn should be allowed to die with his or her family in a private, quiet space. Respectful, culturally sensitive and compassionate bereavement care, including psychological and spiritual support after a newborn death or stillbirth, reduces negative emotional, psychological and social effects for parents and staff [32]. Steps to create or preserve memories are important and should be culturally appropriate.

### Which systems do we need to transform?

Most approaches to improving survival have focused on single interventions. However, most small and sick newborns have more than one problem and therefore require multiple interventions. The delivery of the intervention package outlined above depends on infrastructure, human resources, a bundle of devices and individual level data in information systems to track progress. This health systems transformation is outlined step by step below. Here we focus on the need to link referral and follow-up care systems.

#### Referral systems

Health facilities providing care for newborns require functional referral systems between different levels of the health system, and transport systems to and from the home, promoting non-separation of mother and newborn where possible. Not all complications can be predicted before birth: even with high- quality antenatal and obstetric care, some newborns will require inpatient care unexpectedly. The newborn’s survival depends on speed – minutes count. Each facility should have a clear written policy describing their level of care, including admission and discharge policies, and a written referral plan to guide cases in which a higher or lower level of care (i.e., down-referral) is needed. The policies should also emphasise the importance of returning every newborn and their family to their local facility as soon as appropriate.

#### Follow-up care systems

Discharge planning with parents is essential to ensure their confidence for nurturing care at home. It is necessary to think beyond mere survival to enable babies to thrive post discharge. Follow-up care from trained health-care workers is needed to monitor the baby’s condition, support the family in KMC and nurturing care, and refer in the event of new danger signs or complications. Systems for retinopathy of prematurity screening, follow-up for small babies receiving oxygen therapy, and adequate neurodevelopmental follow-up including referral for early intervention, need to be embedded in follow-up care planning and decision making.

### How to implement faster for small and sick newborn care?

**Fig. 5:**
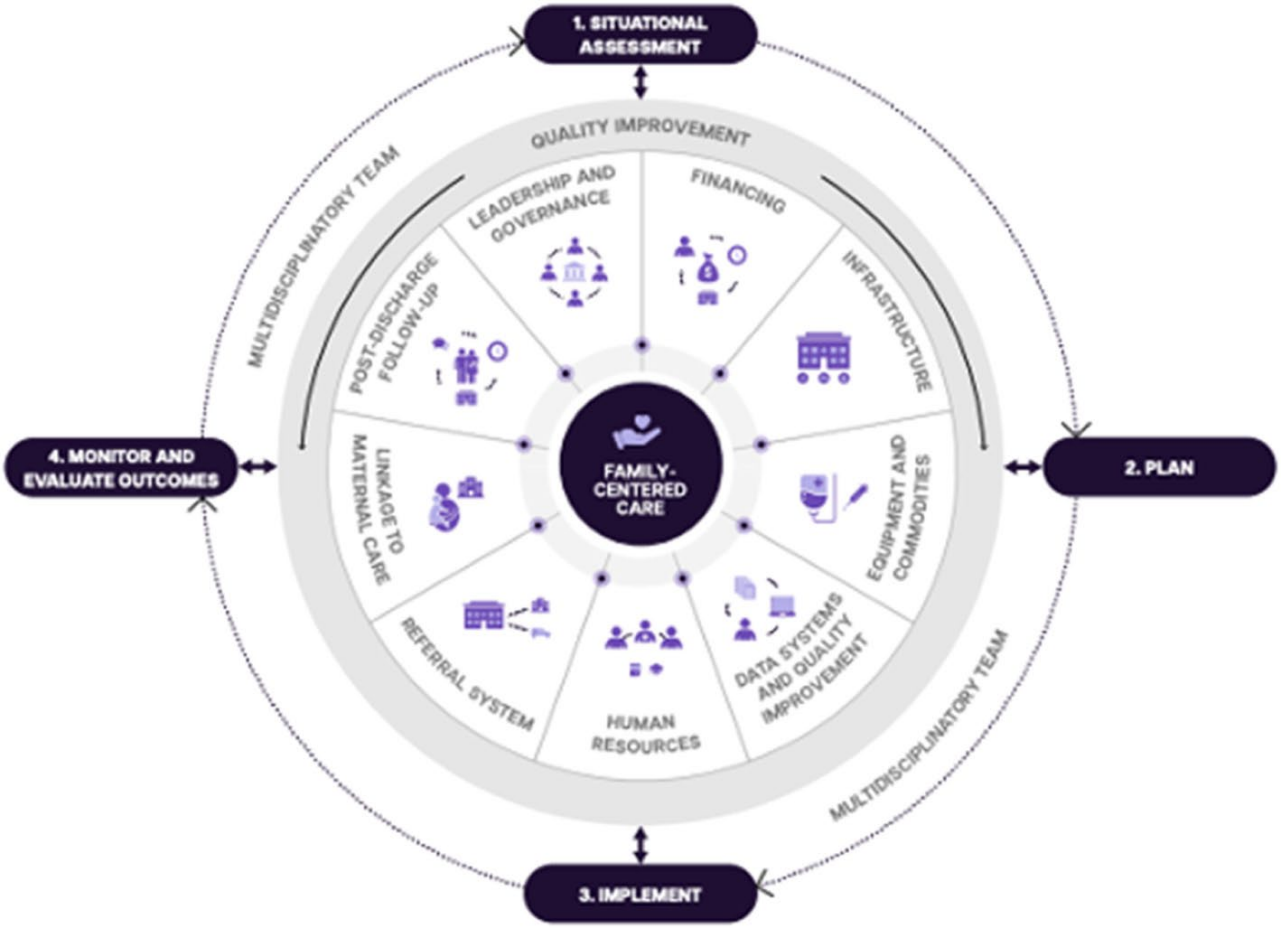
Implementation of small and sick newborn care, with the baby and their family at the centre *Four steps adapted from Knippenberg *et al*. *[55]*. Wheel adapted from *[50]* and the WHO-UNICEF ten core component model for small and sick newborn care scale-up *[34]

Implementing SSNC involves four steps (Fig. 5). Each step requires collaboration by a multidisciplinary team (MDT) focused on each of the ten WHO-UNICEF core components for scaling up care for small and sick newborns at the country level (seven of which are adapted from the WHO health system building blocks) [33]. These components are compatible with components highlighted in the Survive and Thrive report, and with WHO people-centred care [1]. Such MDTs require strong political leadership and commitment, for example from national ministries of health, but multistakeholder input is also needed.

#### Step 1: Situational assessment

**Fig. 6:**
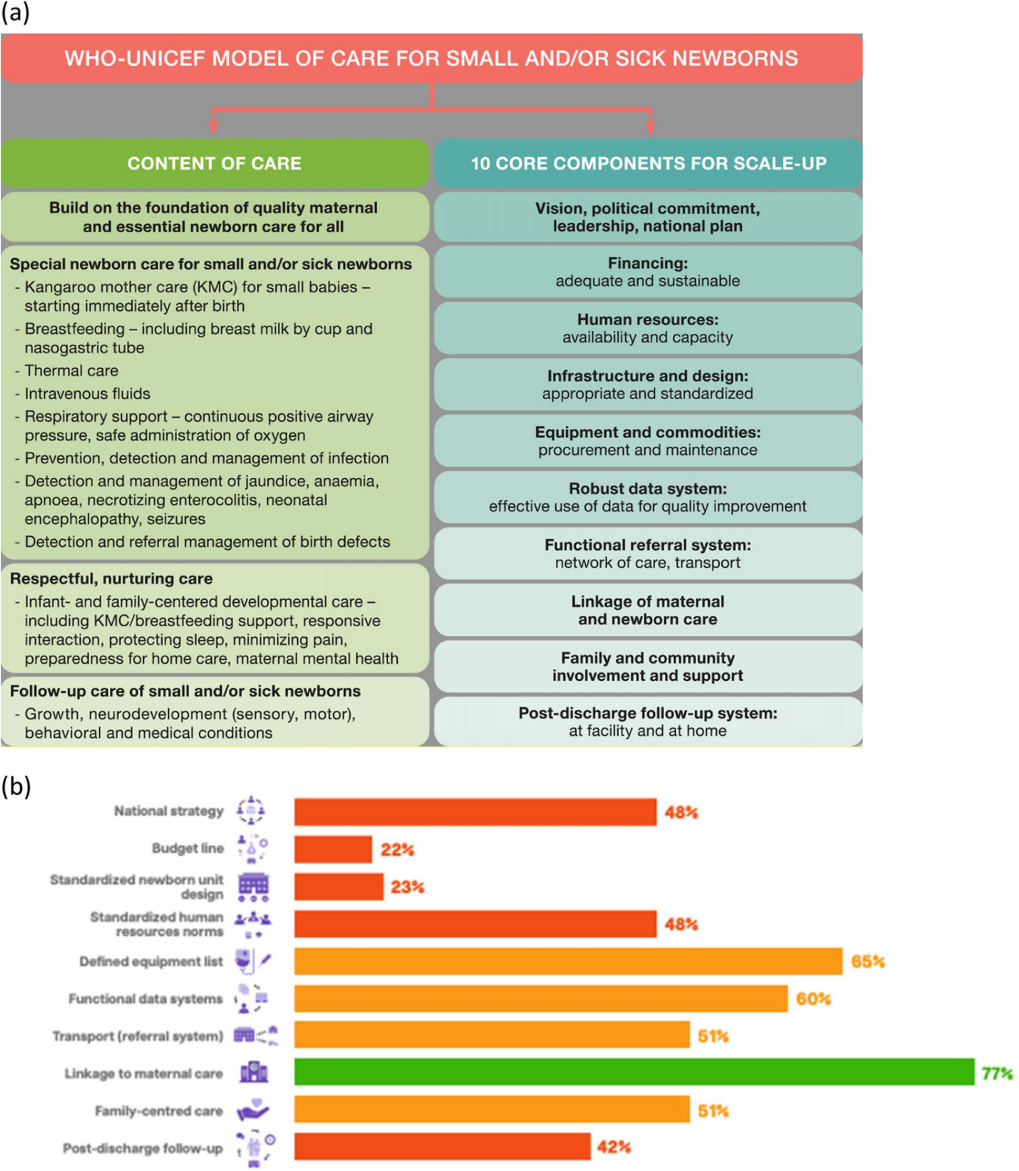
**a** WHO/UNICEF model of care for small and/or sick newborns (**b**) Status of the WHO/UNICEF ten core component model for scaling up small and sick newborn care in 106 countries, based on ENAP/EPMM tracking tool *Note: N* = *106 countries.Data courtesy of UNICEF; Adapted from*[6]

To make informed decisions to improve the quality of newborn care, data are needed on the status of care at the national, subnational and district levels. ENAP coverage target 4 aims for 80% of districts (or equivalent subnational unit) to have at least one level-2 inpatient unit to care for small and sick newborns, with respiratory support including provision of CPAP. Tracking coverage of ENAP target 4 requires capturing the number of districts providing SSNC level-2 and also taking into account equitable distribution. Health facility assessment data should also be collected for each unit providing care, including availability of essential medicines, equipment and staff. Figure 6 depicts situational assessment of 106 countries according to the ten core components for SSNC [34]. The analysis should inform the planning of programmes aiming to improve inpatient care provision, including dedicated budget lines and financing which scored the lowest among all components.

#### Step 2: Plan and budget

Having one national phased implementation plan is effective for countries to reach every district, or equivalent subnational unit. The plan should be developed with key stakeholders, including national and subnational governments, healthcare providers and community members, based on data and the unique needs and challenges. The plan should include a focus on both coverage and quality, through focused targets and actions involving implementers who provide daily care to newborns and their families.

Data should be used to determine where investment is most needed, such as new infrastructure, or increasing numbers and the capacity of health workers, or ensuring availability of essential medicines and supplies. Using data to inform investment decisions enables the allocation of resources in a targeted and effective manner to improve outcomes for small and sick newborns.

Using informed decisions to achieve effective resource allocation also strengthens the case for investment in newborn care. An example of a data-based investment case driving change for SSNC comes from the United Republic of Tanzania [35]. The government led change by including SSNC targets in its National One Plan III, requiring additional investment. The Ministry of Health spearheaded a five-step investment case, building on national policy frameworks and guidelines. Tanzania aims to have functional SSNC level-2 care units in 146 district hospitals and all 25 regional referral hospitals. Secondly, the potential impact of scaling up SSNC was estimated using the Lives Saved Tool for 2025 and 2030, showing that high coverage of SSNC could put the country on track for SDG 3.2 and save an estimated cumulative 79,900 newborn lives by 2030. Thirdly, set-up and running costs were estimated using an activity-based-costing approach and the NEST360 Device Planning and Costing tool [34] (available open access). Set-up costs were estimated at either US$ 112 million (scenario A: all neonatal units built new) or US$ 65 million (scenario B: 50% neonatal units built new, 50% renovated/repurposed), driven by infrastructure. Running costs were estimated at US$ 54 million (scenario A) or US$ 25 million (scenario B), driven by the human resources gap. Even with all neonatal units built new (scenario A) this would be achievable with a 2.6% increase in government health expenditure. Fourth, return on investment was estimated. The investment case in the United Republic of Tanzania is convincing, with a potential return on investment of US$ 7–12 per US$ 1 invested in SSNC. The government has committed additional resources and is mobilising more. And fifth, potential financing opportunities were identified and targeted, with more than US$ 30 million funding allocated from various sources across government, including the president’s office. The five-step process and tools may be useful for other countries to adapt and further refine [35].

#### Step 3: Implement

To effectively implement high-quality care for small and sick newborns in hospital, several key ingredients are necessary.

##### Right place

It is essential that newborns are treated at the right level of care, which requires a functional and effective referral system, and clear, consistent criteria to determine the level of care required for each baby. This is especially important in humanitarian settings, where access to care can be limited. The COVID-19 pandemic highlighted the opportunity for enhanced facility-based newborn care, as investments have been made in staff training, supply chain (including oxygen), data systems and transportation facilities. Often in resource-limited settings the newborn unit is a small, repurposed room, which becomes overcrowded, with limited infection prevention facilities and lacking space for families to be part of care. Countries that have set out standard neonatal unit floor plans such as India [36], Tanzania [35] and Malawi [37] have been able to raise funding more quickly and move faster on improving infrastructure.

##### Right devices, drugs and diagnostics

The right medical supplies and devices are a prerequisite for high-quality care for small and sick newborns. WHO has set standards for detecting and managing clinical syndromes that require specialised medical supplies, devices and diagnostics [8]. This includes ensuring that the right equipment and medications are available at the appropriate level of care, that these are in good working order, and that health-care workers are trained in their correct use. Medications such as caffeine citrate (recommended by the WHO to treat and prevent apnoea of prematurity) is still not routinely available in many LMICs [38]. Low-cost technologies that positively affect survival rates are increasingly available, including those adapted for low-resource settings (e.g., bubble CPAP, point-of-care diagnostic tools including bilirubinometers and haemoglobinometers, etc.). Planning the right devices and numbers for a SSNC unit can be helped by user-designed tools [39]. However, detection of neonatal sepsis, an important cause of neonatal mortality, is a major gap, especially in LMICs [40]. A revised Maternal and Newborn Health commodities list from WHO is in pipeline, which will include listing of all commodities needed to deliver effective level-2 care, and will be of help for countries to guide procurement.

##### Right people

Across all shifts, 24/7**.** Strengthening the health workforce is crucial, and should include valuing systems, mentorship and opportunities to combat attrition. Policymakers, implementers, managers, health-care workers, biomedical engineers and families all play important roles in improving the implementation of evidence-based interventions for SSNC level-2 (Table 1). Skilled health-care workers must be trained to provide high-quality, respectful care [17]. A multidisciplinary team is essential, including neonatal nurses, clinicians, biomedical engineers, laboratory staff, procurement staff and managers, all working together to sustain high-quality SSNC at the institutional level. Data clerks are often overlooked, but also play a critical role in maintaining the quality of care. Many LMICs lack a skilled neonatal workforce which is essential to provide quality care. Retention of staff is challenging, and rotation of skilled staff members affects continuity of care and sustained skill within a neonatal unit. Human resource strategies to improve newborn care in health facilities in LMICs urgently need to be adopted, including mentorship opportunities, creation of enabling and supportive environments for healthcare workers, and systems for supervision and feedback [41].

**Table 1:** The right people are needed to provide small and sick newborn care

**Caregivers especially the mother**	Zero separation is a core principle. Separation affects KMC, breastfeeding, family bonding and mental health [53] COVID-19 exacerbated chronic separation policies in many neonatal units worldwide, where parents often feel more like visitors than parents
Family and communities	Community care is an important part of the continuum of care for mothers and newborns, especially in hard- to-serve populations, such as those that are rural or post-conflict. Communities and families must be involved when implementing care for babies that are small and sick [1]
Health-care providers	As of 2020, there was a global shortage of 15 million health-care providers, primarily in LMICs where the burden of neonatal mortality is greatest [54]. Chronic shortages of skilled providers, especially nurses and biomedical engineers, were exacerbated by the COVID-19 pandemic. Inexperience, rotation and attrition are common challenges faced by facilities across all income settings and need to be directly addressed
Country governments and leaders	Strong leadership and governance are at the heart of the process. Government commitment is needed to develop national plans for newborn health, with a dedicated budget line. Collaboration and coordination are needed across governmental ministries, including health, education and transport, as these bodies share responsibilities within an integrated model of health service delivery

CPAP, a non-invasive mode of ventilation used in preterm born neonates with respiratory distress syndrome, has been used since the early 1990s to decrease mortality by up to 50% if implemented with high quality. Table 2 provides an example of the people, place, devices and drugs needed to implement CPAP in low-resource settings.

**Table 2:** The right place, device, drugs and people to improve use of CPAP in lower income settings

Right place	Right device/drugs	Right people
**Space** with oxygen and air supply; capacity to blend oxygen and air is critical to prevent retinopathy of prematurity **Systems** for transport and referral **Strong** essential care package, including resuscitation, thermal care, and infection prevention and control policy	**Sell** as a package of care, not a stand-alone intervention, to enable upstream factors in antenatal care to be addressed which could delay or prevent preterm labour and delivery **Sustainability factors**, to be included from the onset when the CPAP roll-out is planned, include: • financing • oxygen and air supply • human resources • water and electricity **Steroids antenatally**: new evidence shows that any steroid before delivery is better than none. Preterm labour protocols should be available and implemented appropriately and safely	**Staff**, including biomedical and maintenance, infection prevention and control **Senior leadership** buy-in is essential to allow ongoing financial support for consumables and equipment maintenance and repair **Support to staff**: once implemented at facility, provide outreach and support to brief staff, provide regular monitoring and evaluation, especially to discuss successes and failures of initiating CPAP

Successful implementation and roll-out depends on addressing fear of failure, as not every baby survives, and the right patient is critical for success. Facility readiness for implementation must be assessed *before* implementing CPAP and incorporating a feedback loop to debrief staff on the quality of CPAP, and discuss early CPAP success and failures, is important for improving CPAP provision at facility level. An example of CPAP success was seen in South Africa, where a 20% reduction in deaths due to respiratory distress syndrome was attributed largely to use of CPAP at secondary level facilities [42, 43].

#### Step 4: Monitoring and evaluation for quality improvement

Countries need responsive measurement linked to quality improvement systems. Monitoring coverage, quality and mortality outcomes is foundational for quality improvement. This depends on having reliable data that is comparable across neonatal units and ideally across countries, embedded in national systems. Data alone cannot change outcomes and must be used and acted on regularly by health system managers, health-care providers and community members if systems change is to occur. Utilising data, linked with mentorship, can transform clinical care pathways and improve outcomes. Core metrics from ENAP for assessing the impact, progress and quality of SSNC delivered are detailed in Table 3 [44]. Learning from India, which was one of the initial countries to have an online, real-time data base for SSNC units, shows it is possible to develop such systems at scale and use it for regular monitoring of performance, prioritising supportive supervision and quality improvement [36]. Another example of a multi-country, parsimonious neonatal inpatient dataset co-designed with government is with NEST360, at scale in Malawi and used in four other countries with live dashboards for improving quality of care such as KMC and CPAP coverage [45].

**Table 3:** Core metrics for impact, progress, and quality of small and sick newborn care Implementers and planners must focus on outcome indicators for impact to be tracked against 2030 and other global or national targets, noting other metrics are important for driving progress and quality

**Outcome indicators**	Stillbirth rate Neonatal mortality rate Low birthweight and preterm birth rate
**Coverage indicators**	Percentage of facility births, Percentage of districts with an obstetric care unit, Percentage of districts with a SSNC level-2 unit
**Service readiness indicators**	Health facility assessments for maternity care and for SSNC including scoring over time for infrastructure, human resources, devices etc Nurses on neonatal ward day and night per admitted neonates (nurse ratio)
**Quality of care indicators**^a^	Use of KMC for all admitted neonates under 2500 g, sub-divided for those under 2000 g (with additional markers of quality for KMC e.g., duration) Use of CPAP for all admitted eligible neonates (with additional markers of quality for CPAP, e.g., time of starting) Antibiotic treatment for all neonates with clinical diagnosis of sepsis or meningitis (with quality indicators e.g., use of blood culture)
**Process indicators**	Frequency of neonatal/perinatal audits, documentation on use of data for change
**Investment indicators**	Governmental budget lines for child/newborn health, expenditure of official development assistance Financial protection for maternal and neonatal care, including out-of-pocket payments

^a^It is critical to address the lack of indicators for perceived quality of care by families

Given the risk of infections for vulnerable newborns, special attention is needed to enhance infection detection and response including sentinel sites to track antimicrobial resistance. Improved tracking and surveillance is needed to implement effective antimicrobial stewardship programmes, part of the WHO Global Action Plan for Antimicrobial Resistance [46].

## PIVOTS

### Pivot 1: Invest more ambitiously for high returns

Investment in SSNC is a critical need that requires bold action and collaboration from governments, investors and other stakeholders. To achieve better outcomes for newborns, it is essential to set ambitious planning targets, aiming to reach over 80% of districts with at least one functional SSNC unit, aligning with the ENAP coverage target 4 [7]. Engaging a wide audience, including high-level political leaders, with a practical and results-oriented approach is crucial for success [47]. Data should be leveraged at global, national, and local levels to build a strong case for investment, emphasising how transforming newborn care can improve human capital and drive economic development. Addressing cost barriers is vital, including ensuring maternal and newborn care are covered by insurance, that SSNC interventions are recognised under universal health coverage schemes, and reducing out-of-pocket expenses to promote equitable access to high-quality care. Universal health coverage plays a key role in ensuring the most vulnerable women and newborns have access to the care they need, reducing health inequities. Additionally, social protection is essential for marginalised groups, particularly those in the informal sector, who are often excluded from traditional health insurance schemes.

### Pivot 2: Implement high-quality, family-centred care

The urgent need to implement high-quality SSNC affects all stakeholders, including families, communities, healthcare workers, and governments. It is essential to provide evidence-based, family-centred SSNC at level 2, ensuring it is both high-quality and accessible without major direct or indirect costs. Facilities must have adequate space for respectful care, sufficient numbers of skilled healthcare providers, and appropriate devices available at all hours. Trained and motivated neonatal nurses are key to delivering quality care in partnership with families. Additionally, newborn care should be integrated with both maternity and child health services to ensure comprehensive, coordinated care for mothers and newborns. Creative solutions are also needed to optimise space, allowing mothers and newborns to stay together despite common spatial constraints.

### Pivot 3: Innovate through multi-country learning

Over the next decade, greater collaborative multi-country learning is needed to share existing tools and develop or improve technologies and systems innovations for SSNC in all contexts. Table 4 highlights key innovations required across the ten core components for scaling up SSNC in countries, including low-cost technology options that are purposed for low-resource settings and resilient to climate shifts [48, 49]. Climate is a growing threat to health globally; small and sick newborns are particularly vulnerable.

**Table 4:** Innovating for progress in small and sick newborn care

10 core components for scaling up SSNC in countries	Innovations required
Family-centred care	● Innovations to reduce separation of sick newborns and their caregivers ● Operationalising family centred care ● Social innovations to foster family and community engagement
Leadership and governance	● Governance and accountability monitoring and response units allowing district and subdistrict governance mechanisms ● Hospital governance with improved linkage to maternal and neonatal care
Financing	● National investment cases for SSNC ● Innovative financing schemes for SSNC, e.g. blended financing, intersectoral financing [24]
Human resources	● Increasing ratios of nurses, optimal ratios by case mix/context ● Digital training approaches for neonatal nurses ● Innovative education and training approaches that involve less time off the ward ● Mentoring systems that change quality of care and are sustainable
Infrastructure	● Operationalising Maternal and newborn intensive care units ● More data to inform SSNC unit standardised floor plans, power specifications etc ● Costed floor plans, adapted for context
Medical supplies and devices, including equipment and commodities	● Local manufacturers and procurement systems ● New antibiotics ● Lower-cost, robust devices including for CPAP ● Simplified blood culture processes, suitable for low- and middle-income countries ● Outbreak response triggers ● Innovative diagnostics especially sepsis point-of-care tests
Robust data systems	● Integrate maternal, newborn and SSNC data within District Health Information System (DHIS) ● Operationalizing and using Facility-level data dashboards ● Better capture of SSN interventions especially quality ● Feasible measures of family perceptions of quality of care
Referral systems	● Use of telemedicine technologies ● Innovations for referral systems e.g., communications and GIS mapping ● Lower cost emergency transport systems
Linkage with high-quality maternal care	● Linked newborn-maternal records
Post-discharge follow-up systems	● Feasible package for follow-up care of at-risk newborns ● Innovative ways to increase awareness and education for caregivers e.g., mobile messaging ● Data linkage from maternity, to neonatal to child

*Adapted from *[6]

To develop locally appropriate, robust, and economical devices requires implementation research and usability testing to ensure their feasibility in various contexts [48, 49]. Establishing equitable partnerships between public and private organisations, communities, and researchers is essential for ensuring that innovations reach those who need them most. Additionally, strengthening learning networks and fostering knowledge exchange will enable faster progress and help protect the gains made in newborn survival over the past decade.

Large online platforms can help share tools and learning – for example www.newborntoolkit.org which has almost 1000 tools in 11 languages and had > 50,000 users from 170 countries in the last 12 months [50].

### Pivot 4: Integrate family- centred care and follow-up for developmental outcomes into systems

Operationalising follow-up care is a critical component for preventing or managing morbidity associated with premature birth and other neonatal conditions. For example, increasing data show that after neonatal sepsis, children are at long term risk of impairment, as well as after well-known high-risk conditions such as meningitis, hypoxic ischaemic encephalopathy, necrotising enterocolitis, or severe jaundice [51]. To address this, efforts should focus on family-centred systems that meet the needs of both mothers and newborns—a shift from provider-centred care [17]. This transformation includes providing culturally appropriate care with space for families to stay close to their sick newborns, and carry out practical needs such as washing and eating. Additionally, integrating follow-up into existing systems is essential, incorporating regular check-ups, screenings including for vision and hearing, and support for healthy growth and development. Ensuring financial protection for families of small and sick newborns is also a priority to support their care needs, such as parental leave and minimising out of pocket payments [52].

## Conclusion

Neonatal conditions are the leading cause of lost human capital and have been since 1990 [19]. Each year 2.3 million newborn lives are lost. The tens of millions of small and sick newborns needing inpatient care annually are an opportunity for impact. Action is needed to address the coverage, equity, and quality gaps worldwide. To ensure every small and sick newborn everywhere receives the care they need, ambitious investment in newborn care is essential. Multi-country learning networks and innovations are critical to overcoming shared challenges. High-quality, family-centred SSNC, if implemented, can transform the survival and developmental outcomes of newborns, and also lift the development of nations.

## Data Availability

All data is available in the paper or in supplementary files. Additional information is available at www.borntoosoonaction.org

## Abbreviations

CPAP: Continuous positive airway pressure
ENAP: Every Newborn Action Plan
EPMM: Ending Preventable Maternal Mortality
EWENE: Every Woman Every Newborn Everywhere
KMC: Kangaroo mother care
LMICs: Low- and middle-income countries
MDT: Multidisciplinary team
MNCH: Maternal, newborn, and child health
NEST360: Newborn Essential Solutions and Technologies
NMR: Neonatal mortality rate
SDG: Sustainable development Goal
SSNC: Small and sick newborn care
UN: United Nations
UNICEF: United Nations International Children's Emergency Fund
WHO: World Health Organization
